# Chemical Profiling and Bioactivity Assessment of *Helichrysum italicum* (Roth) G. Don. Essential Oil: Exploring Pure Compounds and Synergistic Combinations

**DOI:** 10.3390/molecules28145299

**Published:** 2023-07-09

**Authors:** Mateo Glumac, Zvonimir Jažo, Vlatka Paštar, Anja Golemac, Vedrana Čikeš Čulić, Sanida Bektić, Mila Radan, Ivana Carev

**Affiliations:** 1 Department of Biochemistry, Faculty of Chemistry and Technology, University of Split, Ruđera Boškovića 35, 21000 Split, Croatia; mateo.glumac@mefst.hr (M.G.); zvonimir.jazo@gmail.com (Z.J.); mila.radan@ktf-split.hr (M.R.); 2 School of Medicine, University of Split, Šoltanska 2, 21000 Split, Croatia; vedrana.cikes.culic@mefst.hr; 3 Regional Laboratory Split, Croatian Veterinary Institute, Poljička Cesta 33, 21000 Split, Croatia; 4 Mediterranean Institute for Life Science, Meštrovićevo Šetalište 45, 21000 Split, Croatia; vlatka.pastar@gmail.com (V.P.); anja.golemac@medils.hr (A.G.); 5 Faculty of Sciences, University of Tuzla, Univerzitetska 4, 75 000 Tuzla, Bosnia and Herzegovina; sanida.osmanovic@untz.ba; 6 Faculty of Science, University of Split, Ruđera Boškovića 33, 21000 Split, Croatia; 7 NAOS Institute of Life Science, 355, Rue Pierre-Simon Laplace, 13290 Aix, France

**Keywords:** breast cancer cell lines, MDA-MB-231, human dermal fibroblast, HDF, lung cell lines, BEAS-2B, MTS and MTT assay, *Helichrysum italicum* (Roth) G. Don., *immortelle*, SEM

## Abstract

*Helichrysum italicum* (Roth) G. Don., immortelle, is a plant species used in ethnomedicine and the food industry as a spice added to food, beverages, and bakery products. It has been shown to possess various biological activities, such as antioxidant and antibacterial activity, making it useful as a natural preservative. We investigated the phytochemical profile and biological activity of *H. italicum* essential oils from wild-grown plant material collected from natural habitats in the Republic of Croatia and Bosnia and Herzegovina. Using high-resolution scanning electron microscopy (SEM), a visual investigation of plant organs (stem, leaf, and flower) was performed, confirming the presence of essential oil reservoirs on the surface of all examined plant organs. Essential oils were isolated by hydrodistillation in the Clevenger apparatus. The chemical composition of the essential oils was determined using the GC-MS analytical technique. Cytotoxic activity tests were performed in vitro on three cell lines: skin (fibroblast), lung, and breast cancer. Using statistical tools, the synergistic and selective effects of *H. italicum* essential oil on healthy and tumor cells were correlated to chemical composition and cytotoxic activity. The synergistic and antagonistic effects of *H. italicum* essential oil’s individual components were simulated by testing pure compounds and their mixture of cytotoxic activity on fibroblasts and breast cancer cells. The results confirm that essential oil’s biological activity is much greater than the sum of the effects of its components. The present data are novel contributions to the body of knowledge on the biological activity of this species used in the food industry.

## 1. Introduction

*Helichrysum italicum* (Roth) G. Don., also known as curry plant or immortelle, is a perennial herb from the Asteraceae family. It is native to the Mediterranean region but can also be found in other parts of the world, such as Africa [1,2]. It grows as a 30–70 cm-tall shrub or subshrub. The stems are upright, leafy, and covered with hairs. The lower and middle leaves are elongated, linear, blunt-tipped, hairy, green on the upper side, and silvery on the underside. The upper side of the leaves is rarely glandular, while the bottom is tomentose (covered with woolly hairs) and glandular. The inflorescence is a head, 4.5–62 × 3–80 mm in diameter, with 2 to 120 intensely yellow flower heads. The head size is 4–6.5 × 2–5 mm, cylindrical to narrowly bell-shaped [3,4]. Mediterranean *Helichrysum* blooms from June (May) to August (September) [3,5].

In Croatia, *H. italicum* grows alongside the Adriatic coast, on islands, and in the Dalmatian hinterland. Being a xerophytic plant, it can grow in dry, sandy, and rocky areas, usually on carbonate and skeletal soils [1,4,6,7].

In general, essential oils are more or less complex mixtures of compounds due to their beneficial properties and can be applied for various purposes such as antimicrobial and anti-oxidative action, to preserve food, and as enzyme inhibitors [8,9,10,11]. Chemical compounds isolated from plants make up most of medicine’s medicinal substances. Some of the most important chemotherapeutic agents in current use, such as vincristine, vinblastine, taxol, irinotecan, etoposide, and paclitaxel, were originally isolated from plant material. Although essential oils have been in use for thousands of years, they have only recently begun to be considered for this purpose, so there currently need to be registered EO compounds in use for this purpose. Numerous aesthetic oils and individual compounds are successful in the fight against cancer. Some perillyl alcohol compounds have already passed phase I and phase II clinical trials for cancer treatment, while even some EOs are part of clinical trials, so it can be expected in the near future that volatile compounds will be used for this application [12].

*Helichrysum italicum* has been used for centuries in traditional medicine for its various healing properties. It has been proven to have anti-inflammatory, antioxidant, and antimicrobial effects and has been used to treat various conditions, such as wounds, bruises, and respiratory problems. In recent years, *H. italicum* has gained popularity in the skincare industry for its ability to regenerate skin cells and improve skin texture. It is often used in anti-aging products and can help reduce the appearance of fine lines and wrinkles. Overall, *H. italicum* is a versatile herb with various uses and potential health benefits [1,2,3,13,14,15,16].

Along with their health benefits, *H. italicum* flowers can be used for seasoning, flavoring food, and as natural food preservatives. It was shown that *H. italicum* curry-like aroma flowers are rich in carotenoids, nutritionally valuable long-chain PUFAs, calcium, potassium, iron, zinc, and other trace elements [17].

*Helichrysum italicum* is commonly used to isolate its essential oil (EO). The EO of *H. italicum* was the subject of a few studies, which have determined a large variability in its chemical composition, attributed to the influence of geographical origin, environmental factors, growth phase, and genotype [18,19,20]. The most common compounds were shown to be: *α*-pinene, limonene, *α*-terpineol, italicene, *ar*-curcumene, nerol, and selinenes [2,3,4,5,18,19,21,22,23,24,25,26,27,28]. Three groups of chemotypes can be found in *H. italicum* EO: (I) nerol and its esters—the most commonly described in the literature; (II) *α*- and *β*-selinene; and (III) *γ*-curcumene [5,27,29].

Several authors have found a correlation between the number of peltate trichomes and essential oil yield in some plant species [30,31]. This inspired us to try to visualize the morphology of the *H. italicum* stem and leaves using SEM. Our study investigated the chemical composition using GC/MS of nine *H. italicum* essential oils collected along Croatia’s coast. We have investigated the relationship between the chemical composition of nine *H. italicum* EOs and their cytotoxic properties in healthy and cancerous cell lines. We applied several chemical classification strategies to characterize bioactive compounds in complex EO mixtures. This prompted us to test individual compounds found in *H. italicum* EO and their mixtures to demonstrate the complexity of EO’s biological activity.

## 2. Results and Discussion

### 2.1. Morphological Study of Aerial Parts of the Plant

**Figure 1:**
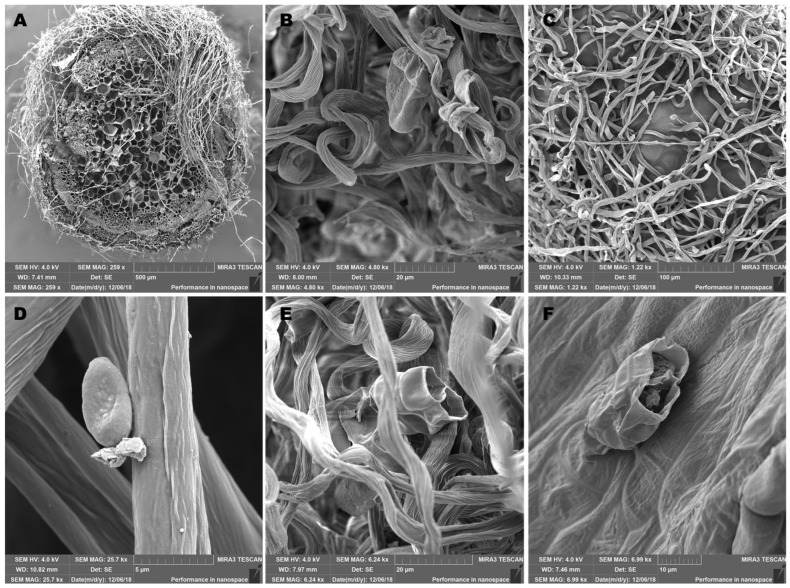
The surface of plant organs (stem, leaf, and flower) of Mediterranean *Helichrysum* (*H. italicum* (Roth.) G. Don) created using a scanning electron microscope (SEM). (**A**) Cross-section of the stem-top view of *H. italicum* (**B**) Steam gland located on the hairs of *H. italicum*. (**C**) The surface of a leaf of *H. italicum*. (**D**) Glandular trichome on the leaf surface of *H. italicum* (**E**) Steam: the gland in the middle contains a biological barrier that divides the interior into two compartments. (**F**) Essential oil reservoir on the surface of the flower of *H. italicum*.

Scanning electron microscopy (SEM) of aerial parts of the *H. italicum* plant material was performed to detect glandular trichomes where the synthesis and accumulation of essential oils occur and to determine which parts of the *H. italicum* plant should be used to isolate EOs. Although glandular trichomes of several *Helichrysum* species have been described [32,33,34], such reports supported with SEM imaging are rare, considering the size of the *Helichrysum* genus worldwide. To contribute to the available knowledge, we studied the glandular structures of *H. italicum* (Roth.) G. Don. The surface of the aerial parts of *H. italicum* (stem, leaf, and flower) observed using SEM is shown in Figure 1.

The presented images show the actual state of the plant immediately before the distillation process. The surface examination was carried out with dry plant material. The surface of the aerial parts is covered with hairy, non-glandular trichomes that protect the plant from low temperatures, sudden drying and water loss, and harmful UV-B radiation. We identified the presence of the glandular hairs in three zones: basal, middle, and apical zones, located on petals, bracts, leaves, and stems (Figure 1), as described before [35]. The glandular hairs that secrete essential oil belong to the same morphological type and are comprised of 12 cells [35]. We noticed that the glandular trichomes are both localized on the hairs (Figure 1B,D) and on the surface of the plant (Figure 1F). We also noticed that glandular trichomes located on the flowers are not protected by hairy structures and, as such, are more exposed to environmental influences (Figure 1F). The secreted material is stored in the subcuticular space, which is formed by the separation of the cuticle from the outer surface of the cell wall of the head’s top layer and is released by cuticle rupture, as can be seen in Figure 1E,F. Additionally, these two photographs show the biological barrier in the interior of the trichome, which divides it into two compartments. As all aerial parts of the plant contained essential oil reservoirs on the surface, we have used all aerial parts of the *H. italicum* plants for EO extraction.

### 2.2. Chemical Composition of H. italicum Essential Oils

Aerial parts of *H. italicum* collected from eight different locations in the Dalmatia region of Croatia and one location in Bosnia and Herzegovina were subjected to hydrodistillation in a Clevenger apparatus to extract EOs. We have used homogenous collection conditions for EO extraction regarding plant development, environmental factors, and collecting conditions to allow a more consistent comparison of biological activity. The yield of EO was in the range of 0.23–0.46%. We analyzed the chemical composition of the obtained EOs using a GC/MS-coupled system. Forty different chemical compounds were identified in nine samples (Table 1), which comprised 80.28–99.99% of the oils. In samples KO-2, KO-1, PL, MA, LA-Z, and KS, sesquiterpene hydrocarbons (23.8–36.01%) were the most represented group of compounds, while in samples LA-P (33.47%), VI (28.59%), and PT (23.75%), a high proportion of non-terpene compounds was detected. Only three compounds (*α*-pinene, *α*-fenchene, and limonene) were detected, which are classified as monoterpene hydrocarbons and, depending on the sample, make up 6.21–18.56% of the oil. The plant’s picking time explains the high proportion of sesquiterpene compounds. Sesquiterpenes are the dominant group of compounds during the later summer months after the flowering period, while monoterpenes are more represented during the vegetative period [5]. The main compound in EOs of *H. italicum* is neryl acetate, whose amount in the analyzed samples was 4.48–21.36%. Research conducted among EO *H. italicum* producers showed that the proportion of neryl acetate greater than 5% and *α*-pinene less than 25% indicates the high quality of *H. italicum* EOs [36]. In our samples, the proportion of *α*-pinene ranges from 4.01–12.64%; therefore, the analyzed *H. italicum* EOs can be considered of high quality. Only the sample LA-Z contains neryl acetate in a proportion slightly less than 5%. Research has shown that the proportion of neryl acetate in the plant is variable; it increases during the vegetative period and decreases after flowering [5]. In our samples and in EOs of *H. italicum* originating from plants collected throughout Croatia, BiH, and Serbia, the proportion of neryl acetate can be considered equivalent [29,37,38,39]. Higher proportions of neryl acetate (30–45%) were detected in *H. italicum* EOs originating from Corsica and southern Italy [20,40,41]. Other terpene compounds found in all samples are limonene, *β*-selinene, *α*-copaene, italicene, *α*-terpineol, *Ar*-curcumene, and *β*-selinene. According to literature data, nerol is one of the constituents of EO *H. italicum*. We did not include it among the main compounds because it was not detected in all samples (LA-P and LA-Z), which in turn were the only samples containing the sesquiterpene alcohol nerolidol. This could be explained by the specific geographic position of these two locations. The preferred antioxidant compound in *H. italicum* EO is *γ*-curcumen, which is very unstable and quickly transforms into italicene and isoitalicene; under the influence of light, it changes into *α*-curcumen [36,42]. The oils from locations KO-1 (6.85%) and KO-2 (6.82%) can be considered higher quality because they contain a higher proportion of *γ*-curcumen than other samples.

Diketone compounds with the trivial names italidione I, italidione II, and italidione III could also be considered the main compounds because they are represented in all analyzed *H. italicum* EOs, representing 12.4–29.61% of the oil. The presence of italidiones in EO *H. italicum* is also reported in the works of other authors [43,44]. In most samples, the share of derived monoterpenes (8.89–27.06%) is higher than the share of derived sesquiterpenes (2.92–17.17%). The exception is sample LA-Z.

Depending on their chemical composition, *H. italicum* Eos is classified into different chemotype groups. Three chemotypes [5,27,29] with a high proportion of individual compounds are most often mentioned in the literature: (I) nerol and its esters chemotype; (II) *α*- and *β*-selinene chemotype; and (III) *γ*-curcumen chemotype. Most of the Eos of *H. italicum* samples in this work are of chemotype I; the exception is samples LA-P and LA-Z, which are of chemotype II.

### 2.3. The Cytotoxicity of H. italicum Essential Oils

The cytotoxic activity of nine *H. italicum* EOs was evaluated in vitro for 48 h using different cell lines: human dermal fibroblasts (HDF), immortalized lung cells (BEAS-2B), and breast adenocarcinoma cells (MDA-MB-231). For nine EOs tested on three cell lines, IC_50_ values are presented in Table 2.

**Figure 2:**
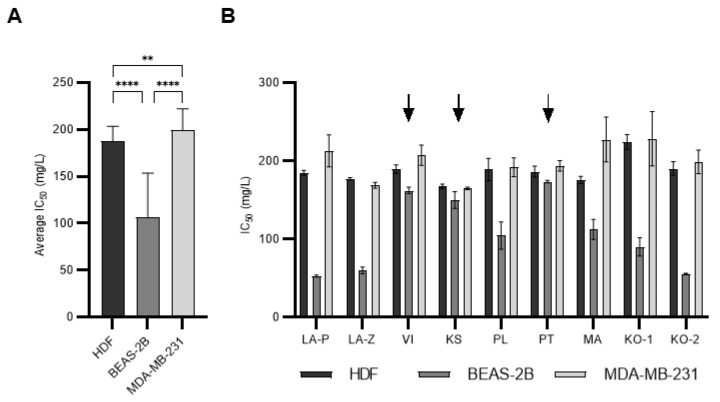
Comparison of cytotoxic effect of *H. italicum* EOs. (**A**) HDF, BEAS-2B, and MDA-MB-231. (**B**) Comparison between average IC_50_ values for tested essential oils on three cell lines. Values were compared by performing a two-way ANOVA test with post hoc Tukey’s corrected multiple comparison tests. Symbols for different test significance levels are assigned as follows: ** for 0.001 ≤ *p* < 0.01, and **** for *p* < 0.0001. All data are presented as mean ± standard deviation (SD). All data are presented as mean ± standard deviation (SD). Arrows indicate EOs with similar cytotoxic effects on all three cell lines.

The effect of the tested oils on cells could be classified according to the criteria of the US National Cancer Institute, which defines four levels of cytotoxic activity [45]. All tested EOs samples showed moderate (IC_50_ = 21–200 mg/L) or weak cytotoxic (IC_50_ = 201–500 mg/L) activity on three tested cell lines. The results showed that the BEAS-2B line was the least resistant cell line to the activity of *H. italicum* Eos, and all samples were moderately cytotoxic. This was confirmed by comparing the average IC_50_ value obtained for each cell line (Figure 2A). Similar results were obtained in a previous study we conducted with another plant species, BEAS-2B, which was also the least resistant cell line to the action of EO [46]. The results also suggested that the MDA-MB-231 cell line was the most resistant to the effects of *H. italicum* EO. Interestingly, three EOs (VI, KS, and PT) showed very similar cytotoxic potential on all three cell lines, which is in contrast to the other EOs (Figure 2B). These results indicate that the cytotoxic potential of EOs, which stems from differences in composition, also depends on the cell type the EO was tested on. Different cell types will respond differently to the same Eos.

### 2.4. Statistical Correlation of Cytotoxicity and Essential Oil Chemical Composition

To better describe the dependence of the cytotoxic activity of *H. italicum* EO on its chemical composition, the identified compounds were classified into several groups defined according to the type of terpene compounds, functional groups, structural characteristics, molecular size (molecular weight), number of oxygen atoms, and number of unsaturated chemical bonds in the molecule (Table S1). According to statistical analysis, *H. italicum* produces EOs rich in terpene compounds of simple structure. Oxygenated compounds compose most of the Eos, and almost all compounds contain at least one unsaturated bond in their structure (Supplementary Materials Figure S1).

Obtained groups were correlated with the IC_50_ value of individual EO by computing Pearson’s correlation coefficients (*r*) and *p* values. A negative correlation coefficient *r* shows that the examined data groups behave inversely proportionally. In the context of our results, *r* < 0 means that a higher proportion of an individual compound or a group of compounds was found in oils with a lower IC_50_ value, suggesting that that group of compounds increases the cytotoxicity of the tested EO and vice versa. Only those results with *p* < 0.05 were considered statistically significant. The agreement of the statistical and experimental results was checked by comparing the samples. The significant results of statistical data processing are shown in Table 3, while the complete analysis is presented in Tables S2 and S3.

After the statistical test, compounds that significantly affected the viability of the HDF cell were terpinene-4-ol, nerol, *γ*-curcumene, and *2*-methylbutyl angelate, showing a positive correlation. This is in accordance with experimental data, where samples KO-1 and KO-2 were the least cytotoxic to the HDF line. They contain the previously mentioned compounds in the highest proportion. The KS sample had the highest cytotoxic activity on the HDF line. Terpinene-*4*-ol and *γ*-curcumene were not detected in that sample, while the proportion of nerol and 2-methylbutyl angelate was less than 1%. Based on the chemical composition and estimated IC_50_ values, it is reasonable to assume that compounds with a positive correlation and a significant *p* value reduce the cytotoxic activity of *H. italicum* EO on dermal fibroblast cells.

We mentioned earlier that the BEAS-2B line is the least resistant to the effects of *H. italicum* EO. After the statistical test, groups of compounds with a negative and significant correlation were observed, indicating greater cytotoxic activity of *H. italicum* EOs on the BEAS-2B line. A good agreement between the experimental and statistical results was observed for *δ*-cadinene, which contributes to greater cytotoxic activity according to several parameters: it is classified as an alkene, its molecular weight is above 200 g/mol, it is built from two cyclic rings, it contains two chemically unsaturated bonds, and it does not contain an oxygen atom. The samples that showed the highest cytotoxic activity on the BEAS-2B line were LA-P, KO-2, and LA-Z. In each of the mentioned samples, one or more groups of compounds with a negative correlation were observed. Compared to other samples, sample LA-Z contains the highest proportion of alkene compounds (*α*-terpineol and *α*-copaene), which were statistically found to increase the cytotoxicity of *H. italicum* EO. The cytotoxic activity of the KO-2 sample could be a consequence of the synergistic action of different groups of compounds with a negative correlation; in this sample, the largest proportion of compounds with a molecular weight greater than 200 g/mol are those compounds that contain two unsaturated chemical bonds in their structure and *δ*-cadinene. Statistically significant compounds with a negative correlation whose molecular mass is greater than 200 g/mol are *α*-copaene and italidione II. The greater cytotoxic activity of *H. italicum* EOs on the BEAS-2B line is also contributed by cyclic compounds made of two rings, and the largest proportion of these compounds was detected in the LA-P sample. A higher cytotoxic activity could be expected for sample PL because it contains the largest proportion of alkenes, the largest proportion of compounds that do not contain an oxygen atom in their structure, and a high proportion of cyclic compounds with two rings. The aforementioned groups of compounds that increase the cytotoxic activity of *H. italicum* EO on BEAS-2B are synergistically opposed by the statistically significant compounds neryl acetate and *ar*-curcumene. Oil samples with a total content of neryl acetate and *ar*-curcumen greater than 19% were less cytotoxic to BEAS-2B. A correlation between experiment and statistics was observed for groups of compounds that contribute to a lower cytotoxic activity of *H. italicum* EO on the BEAS-2B line, namely: derived monoterpenes, esters, aromatic compounds, and compounds whose molecular weight is in the interval of 150–197 g/mol.

By statistical data processing, it was observed that *β*- and *γ*-selinene isomers have a significant and opposite effect on the MDA-MB-231 breast cancer line. The truth of the statistical prediction can be verified by comparing the samples with the highest (LA-Z, KS) and lowest (KO-1, MA) cytotoxic activity on the MDA-MB-231 line. The absence of the *γ*-selinene isomer in KO-1 and MA samples reduced the cytotoxic activity of *H. italicum* EO on the MDA-MB-231 line. In samples LA-Z and KS, the isomers appear as a racemic mixture, and in these two samples, *γ*-selinene was detected in a high proportion. Based on experimental observations, the truth of the statistics and the claim that *β*-selinene does not have a cytotoxic effect on the MDA-MB-231 line, while *γ*-selinene has a more active cytotoxic effect, can be confirmed. The similarity between HDF and the MDA-MB-231 line is manifested in the action of *2*-methylbutyl angelate. This compound is significant for both cell lines. The similarity with the BEAS-2B line is manifested in the action of compounds whose molecular weight is 150–197 g/mol. The samples with the lowest cytotoxic activity on the MDA-MB-231 line contain a higher proportion of compounds with a molecular weight of 150–197 g/mol. Statistical data processing showed that saturated compounds have a significant and negative correlation with the MDA-MB-231 line. The detected saturated compounds are *1,8*-cineole, *endo*-borneol, and viridiflorol. For the three mentioned compounds, antitumor activity was proven in the works of other authors [47,48,49]. In a previous study we conducted with another plant species, we also learned that viridiflorol showed greater cytotoxic activity on the breast cancer line [46]. In this paper, the *p* value for viridiflorol is 0.0543, which is slightly above the significance limit. It should be taken into account that the EO of *H. italicum* is a more complex mixture of compounds compared to other types of EO. The saturated compound viridiflorol was detected in sample KS (1.53%), while *endo*-borneol was detected in sample LA-Z (1.81%). The samples with the highest cytotoxic activity on the MDA-MB-231 line contained the lowest proportion of compounds with three chemically unsaturated bonds. The lower cytotoxic activity of *H. italicum* EO on MDA-MB-231 can probably be explained by the proportion of the statistically significant compound rosifoliol, with positive correlation coefficients calculated. Samples KO-1 (4.67%) and MA (6.24%), which showed the least cytotoxic activity on the MDA-MB-231 line, contain rosifoliol in the largest proportion. Considering that rosifoliol does not fall into any group of compounds for which a statistically significant effect on the MDA-MB-231 line was demonstrated, we assume that its action manifests itself as a synergistic effect.

Nevertheless, despite the statistical correlations, it is necessary to test selected pure compounds in vitro and prove their activity, both as pure compounds and in mixtures, as the results might be surprising [50].

### 2.5. The Cytotoxicity of Individual Compounds and Their Synthetic Mixture

**Figure 3:**
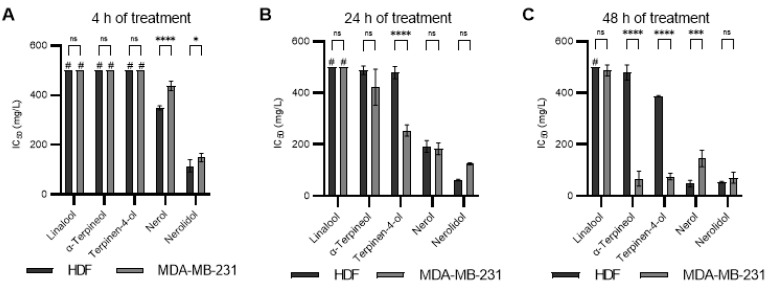
Comparison of individual compound IC_50_ values between two cell lines: (**A**) for 4 h of treatment; (**B**) for 24 h of treatment; (**C**) for 48 h of treatment. Values were compared by performing a two-way ANOVA test with post hoc Šidák corrected multiple comparison tests. Symbols for different test significance levels are assigned as follows: not significant (ns) for *p* ≥ 0.05, * for 0.01 ≤ *p* < 0.05, *** for 0.0001 ≤ *p* < 0.001, and **** for *p* < 0.0001. All data are presented as mean ± standard deviation (SD). (#—IC_50_ were outside of the tested range, excluding values of 500 mg/L).

For further tests, we have chosen five individual compounds: linalool, *α*-terpineol, terpinene-4-ol, nerol, and nerolidol. All chosen compounds align with the preferred chemical profile synthesized by the *H. italicum* plant and are less represented in the studied essential oils. Further, to assess potential selective effects, we have tested individual chemical components from EOs on normal human dermal fibroblasts (HDF) and tumor cell lines (MDA-MB-231), with increasing concentrations of individual chemical compounds detected in the studied *H. italicum* EOs for 4, 24, and 48 h. The results are presented in Figure 3. As shown in Figure 3, all compounds showed cytotoxic potential at least once on either cell line except linalool. Linalool was not cytotoxic for the HDF cell line and only slightly toxic for the MDA-MB-231 cell line at the 48-h time point. Linalool, in high doses, was previously shown to induce apoptosis in MDA-MB-231 [51], where the particular cell line was highly resistant to the treatment. Nerolidol was the most cytotoxic compound in both cell lines and at all time points. This is in accordance with the statistical analysis done in the previous section, where nerolidol showed negative Pearson’s correlation coefficients for both tested cell lines, but the *p*-values were insignificant. This could be explained by only two samples containing nerolidol, reducing the correlation’s significance. Nerolidol induces cell death through disruption of the cell membrane, even in low doses [52]. It also induces multiple types of cell death depending on the applied concentration [53]. Nerol, *α*-terpineol, and terpinene-4-ol show nice time-dependent reductions in IC_50_ values with increasing duration of the treatments. Nerol was the second-most toxic compound. The MDA-MB-231 cell line was more resistant to its effects. Contrary to nerol, *α*-terpineol and terpinene-4-ol were more cytotoxic to the MDA-MB-231 cell line than they were to the HDF cell line. This could be explained by the origin of individual cell lines: the MDA-MB-231 cell line is a tumor cell line, while HDF is a normal cell line. Nerol and *α*-terpineol possessed the same ability to disrupt cell membranes as nerolidol but to a lower extent, which could also be observed in a 4-h treatment [52]. Terpinene-4-ol was shown to induce apoptosis in multiple tumor cell lines [54,55,56].

**Figure 4:**
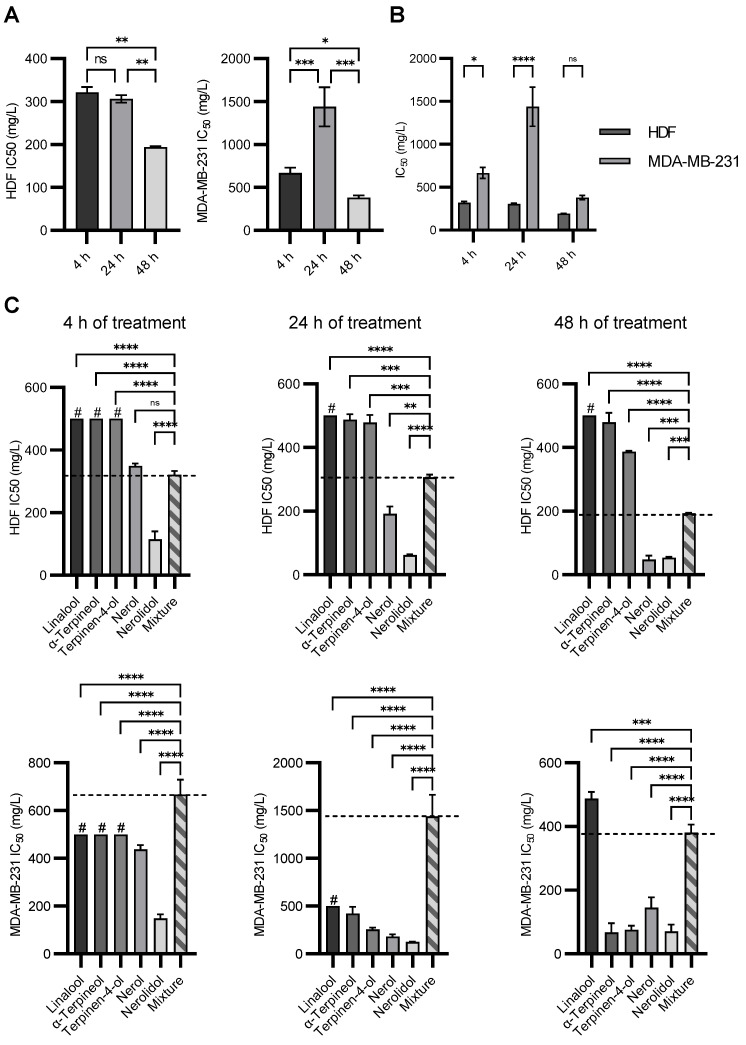
Comparison between the mixture and individual components in HDF and MDA-MB-231 cell lines. (**A**) Time point comparison between IC_50_ values of the mixture for 4, 24, and 48 h of treatment in HDF and MDA-MB-231 cell lines. Values were compared by performing a one-way ANOVA test with post-hoc Tukey-corrected multiple comparison tests. (**B**) Comparison between IC_50_ values from two cell lines treated with the mixture of compounds at different time points. Values were compared by performing a two-way ANOVA test with post hoc Šidák corrected multiple comparison tests. (**C**) Comparison of individual compounds’ IC_50_ values with IC_50_ value of the mixture for 4, 24, and 48-h time points for both cell. lines for 4 h of treatment. Values were compared by performing a one-way ANOVA test with post-hoc Tukey-corrected multiple comparison tests. Symbols for different test significance levels are assigned as follows: not significant (ns) for *p* ≥ 0.05, * for 0.01 ≤ *p* < 0.05, ** for 0.001 ≤ *p* < 0.01, *** for 0.0001 ≤ *p* < 0.001, and **** for *p* < 0.0001. All data are presented as mean ± standard deviation (SD). (#—IC_50_ values were outside of the tested range, excluding values of 500 mg/L.)

Cells were further treated with a range of concentrations of a mixture of all five terpenes to determine how the cells would react to the mixture compared to individual compounds. The data were surprising. The HDF showed the expected time-dependent reduction in IC_50_ value, but the MDA-MB-231 cell line did not (Figure 4A). In addition, the IC_50_ values obtained were much higher than expected. The MDA-MB-231 cell line was more resistant to the mixture compared to the HDF cell line (Figure 4B), albeit it was less resistant to pure compounds (taken together). This suggests the presence of an antagonistic effect between compounds that reduced the cytotoxicity of the mixture. In Figure 4C, we compared the IC_50_ values of individual compounds with the IC_50_ values of their mixtures for both cell lines and for all time points. For the HDF cell line, it is observable that linalool, *α*-terpineol, and terpinene-4-ol have a higher IC_50_ value than the mixture, while nerolidol has a lower IC_50_ value. Nerol showed a similar IC_50_ value at the 4 h time point band, which was lower for subsequent time points. As the mixture contained all five compounds in equal mass concentration as used for individual tests, it could be assumed that the mixture would be equally or more cytotoxic than the most cytotoxic individual compound. As can be seen from the data, this is not the case. Even though the same amount of nerolidol molecules were present, nerolidol in the mixture was less toxic to the cells than on its own. This effect is even more pronounced in the MDA-MB-231 cell line. The mixture of all five compounds could not be considered toxic at the 4 and 24 h time points as it was higher than 500 mg/L [45]. Surprisingly, it even increased at the 24 h time point compared to the 4 h time point. This shows that pure compounds have an antagonistic effect on each other’s cytotoxic potential.

These results could explain why essential oils possess such high IC_50_ values even though they could be composed of highly toxic compounds. This also validates the use of EOs in folk medicine, where the cytotoxic effect of individual compounds is mitigated by the effect of the mixture. This could allow other properties of individual compounds, such as antioxidative activity and the potential for activation of autophagy, to become the major effects of EOs in the cells. Both events would lead to reduced cellular stress and the clearance of accumulated toxins inside the cells [57,58].

Our results show that complex mixtures such as EOs do not affect cells based on the effects of individual compounds but that these effects interact positively or negatively with each other. The effect of synergism and antagonism between bioactive compounds is a very important issue in medicine because it could lead to increased toxicity or inactivation of prescribed drugs [59]. As medical professionals prescribe drugs, these effects could be avoided by carefully selecting bioactive substances. On the other hand, natural products such as EOs are being increasingly consumed [60]. As they could be freely and easily obtained and consumed without restrictions, this could lead to unwanted consequences.

The correlation between cytotoxic activity and compound structure is observed in the example of two individual compounds we tested on HDF and MDA-MB-231. Terpinen-4-ol and α-terpineol are two isomeric compounds that contain the same number of carbon, hydrogen, and oxygen atoms. These two compounds differ in the position of the OH group and the substituent attached to the ring structure. The difference in cytotoxic activity between these two compounds can be explained based on spatial structural differences. Through statistical data processing, it was observed that *β*- and *γ*-selinene have different cytotoxic effects on the MDA-MB-231 line. The two isomeric compounds correlate positively and negatively with the MDA-MB-231 line. The structural characteristics of the compounds themselves probably explain the differences. For a detailed study of the dependence between structure and cytotoxic activity, it is necessary to carry out additional tests. It would be needed to perform the chromatographic analysis with chiral columns capable of separating the two enantiomeric compounds to notice the difference between the amounts of the enantiomeric compounds and detect their presence. Furthermore, additional tests require developing new separation methods for purifying and separating two enantiomeric compounds, which is a very complex task since enantiomers have the same physical properties.

## 3. Materials and Methods

### 3.1. Plant Material Origin

Plant material from grown plants of *H. italicum*, collected from their natural habitat in accordance with legal and botanical regulations for wild plant harvesting, was determined by means of macroscopic traits. Plant material was collected in the coastal area of the Dalmatia region in Croatia in August and September 2018. Voucher specimens (2018_HItalicum_HI) of plant materials used for this study have been deposited, with the date and location of collection, in the herbarium at the Department of Biochemistry, Faculty of Chemistry and Technology, Split, Croatia. On 7 June 2023, the plant name was checked with http://www.theplantlist.org for the last time. The list of location coordinates is shown in Table 4. For the purpose of the research, plant material was air-dried for 15 days at room temperature in the dark. Dried plant material was cut into smaller pieces and used for distillation.

### 3.2. Scanning Electron Microscopy

*Helichrysum italicum* leaves, stems, and flowers were fixed in a standard solution of 25 mM phosphate buffer (pH = 7) with 3% glutaraldehyde (Sigma–Aldrich, Munich, Germany) and 0.01% Tween 20 to observe the epidermal surface. The samples were left in the same fixative solution without Tween 20 for the entire night. FAA fixative, which contains 50% ethanol, 35% distilled water, 10% formaldehyde, and 5% glacial acetic acid, was another fixative employed during the same technique. Samples were rinsed in phosphate buffer three times at 10-min intervals after being fixed at 4 °C overnight. The dehydrated fixed plant material was immersed in absolute ethanol (Sigma–Aldrich, Munich, Germany) and graded into ethanol series (30%, 50%, 70%, and 95%). An K 850 Critical Point Dryer evaporated the ethanol before fixed samples were further dried and sputter-coated with gold (10 nm thickness) using a Q1 50R ES (Quorum, Lewes, UK). An FE-SEM Tescan Mira III (Tescan, Brno, Czech Republic) was used for the observations, which were conducted at a 4 kV accelerating voltage [61,62].

### 3.3. Essential Oil Extraction, Gas Chromatography-Mass Spectrometry (GC-MS) Analyses

The essential oils from the air-dried aerial portions of *H. italicum* were extracted by performing hydrodistillation in a Clevenger apparatus for 3 h. Obtained EOs were kept in a sealed vial at 4 °C until use. A gas chromatograph (model 7890A, Agilent Technologies, Santa Clara, CA, USA) and a selective mass detector (model 5975C, Agilent Technologies, Santa Clara, CA, USA) were used in conjunction to assess the chemical composition of isolated EOs. The injector’s temperature was set at 250 °C. A non-polar, capillary HP-5MS column with a stationary phase made up of 5% phenyl-methylpolysiloxane (30 m × 0.25 mm, layer thickness 0.25 m, Agilent Technologies) was used to separate the chemicals. Helium was used as the carrier gas, with a flow rate of 1 mL/min. The oven temperature was set to isothermal for three minutes at 70 °C, then increased to 200 °C at a rate of three degrees per minute and kept at 200 °C for twenty minutes. The mass detector was set up with the following parameters: an inlet temperature of 280 °C, an ionization energy of 70 eV, and scanning in the mass range of *m*/*z* 30–300.

Individual chemical compounds were identified using retention indices (RI) based on the retention time of *n*-alkanes (C_9_–C_25_), experimental mass spectra, spectra from the commercial Wiley 9 database (Wiley, New York, NY, USA), spectra from an internal database created during previous analyses, and literature retention indices using NIST 2002 (National Institute of Standards and Technology, Gaithersburg, MD, USA) and NIST 2003.

### 3.4. Cytotoxicity Assay

#### 3.4.1. Chemicals and Cell Lines

Dulbecco’s Modified Eagle’s Medium (DMEM, D5796), Dulbecco’s Phosphate Buffered Saline (PBS, D8537), and fetal bovine serum (FBS, F7524) were obtained from Sigma–Aldrich, Munich, Germany. Dimethyl sulfoxide (DMSO, 51779) was obtained from Honeywell, Charlotte, NC, USA. Antibiotic-antimycotic (15240062) and Trypsin-EDTA (0.25%, with phenol red; 25200056) were obtained from Gibco by Life Technologies, Waltham, MA, USA. Reagents used in cytotoxicity assays are MTT (thiazolyl blue tetrazolium bromide), obtained from Cayman Chemicals, Ann Arbor, MI, USA, and MTS (CellTiter 96 Aqueous One Solution Cell Proliferation Assay), obtained from Promega, Madison, Wisconsin. Primary human dermal fibroblasts (HDF) were purchased from Axol, UK. Immortalized lung epithelial cells (BEAS-2B) and breast adenocarcinoma cells (MDA-MB-231) were obtained from the laboratory of the Mediterranean Institute for Life Sciences (MedILS, Split, Croatia).

#### 3.4.2. Measurement of Cytotoxicity of Pure Compounds and Their Mixture with MTT Assay

The cytotoxic effect of pure chemical molecules (linalool, *α*-terpineol, terpinene-4-ol, nerol, and nerolidol) on HDF and MDA-MB-231 cell lines was assessed by a standard MTT assay. Molecule stock solutions were prepared in dimethyl sulfoxide (DMSO). Working solutions for each molecule were prepared by dissolving stock solutions in a growth medium to a final concentration of 10, 125, 250, and 500 mg/L. DMSO was also tested as a control condition at 0.01%, 0.125%, 0.25%, and 0.5%, corresponding to the amounts contained in molecules working solutions. Five pure chemical molecules mentioned above were combined to prepare and test the mixtures. The final concentration of each molecule in the first mixture was 10 mg/L, in the second 125 mg/L, in the third 250 mg/L, and in the fourth mixture 500 mg/L, corresponding to the total concentration of the mixture applied to the cells of 50, 625, 1250, and 2500 mg/L. The treatment was performed 24 h after seeding primary human dermal fibroblasts on 96-well plates. Cells were treated with pure chemical molecules, DMSO, and mixtures for 4, 24, and 48 h. Treated cells were incubated in a humidified 5% CO_2_ incubator at 37 °C. Fresh growth medium containing 0.5 mg/mL MTT working solution was added to the cells and incubated for 4 h at 37 °C in a humidified 5% CO_2_ incubator. Precipitated formazan was dissolved in DMSO, and absorbance was measured at 595 nm using an Infinite M NANO+ Tecan multimode plate reader (Tecan, Austria) for HDF cells, while MTT for breast cancer cells was read at 570 nm in a Biosan plate reader (HiPo MPP-96, Biosan, Latvia).

#### 3.4.3. Measurement of EOs Cytotoxicity with MTS Assays

The cytotoxicity of EOs was determined by performing MTS assays on HDF, BEAS-2B, and MDA-MB-231 cell lines. Cells were grown in DMEM supplemented with 10% FBS and 1% antibiotic-antimycotic. Additionally, 1% sodium pyruvate was added to the medium for growing MDA-MB-231 cells. All cell lines were grown in a humidified 5% CO_2_ incubator at 37 °C.

To prepare stock solutions, EOs were dissolved in DMSO. Further, to prepare EOs working solutions, stock solutions were dissolved in the cell culture growth medium with a concentration range of 125 to 1000 mg/L. The amount of DMSO in working solutions was less than 1%. The EOs treatment was performed 24 h after seeding cells on 96-well plates. The cells were incubated with the treatment for 48 h. After that, treatments were removed, and a fresh growth medium containing MTS reagent was added. The absorbance of samples in biological and technical triplicates was measured at 492 nm with the EnSight (PerkinElmer, Waltham, MA, USA).

### 3.5. Statistical Analysis

The GraphPad Prism software (version 9) was used for the statistical analysis. The software’s built-in equation, [Inhibitor] vs. normalized response—Variable slope, was used to fit normalized cell viability curves. The mean and standard deviation are used to express the calculated IC_50_ values. Shapiro–Wilk normality test results were used to determine the distribution of the data. The distribution of the data was normal (*p* > 0.05). The association between GC/MS data indicating the chemical composition of sage EOs and IC_50_ values evaluating their cytotoxic action was investigated using Pearson’s correlation coefficients (Table 4). R values were utilized to understand the results of the correlation. A higher ingredient concentration in the oil composition reduces the IC_50_ value, indicating that the constituent leads to higher EO toxicity. Values 0 > r > −1 show a negative connection. The positive association between the values of 0 and r indicates that the element contributes to decreased EO toxicity when it is present in higher concentrations in the oil composition (higher IC_50_ value). The stronger the connection, the more the r-value deviates from 0. Only findings with a p-value less than 0.05 were considered significant. Experiments containing two subgrups were analyzed using a two-tailed unpaired *t*-test. Experiments containing three or more subgroups with one variable were analyzed using one-way ANOVA. Post hoc analysis was performed using Tukey’s multiple comparison test between any two groups. For multiple-variable experiments, a two-way ANOVA was performed. Post hoc analysis was performed using Tukey’s or Sidak’s multiple comparison tests. Each presented statistical test was disclosed in the figure or table legends. Only data containing *p* < 0.05 were considered statistically significant. Symbols for different test significance levels are assigned as follows: not significant (ns) for *p* > 0.05, * *p* < 0.05, ** *p* < 0.001, *** *p* < 0.0001, and **** *p* < 0.00001. The data were expressed as mean ± SD. The sample size was n ≥ 3, containing biological replicates.

## 4. Conclusions

We investigated the aerial parts of *H. italicum*. Using SEM, we have determined that stems, flowers, and leaves contain essential oil reservoirs on their surfaces, proving that all areal plant organs are viable sources of EO. Further, we have investigated the chemical composition using GC/MS of nine *H. italicum* essential oils collected along the Croatian coast. The main compound in EOs of *H. italicum* is neryl acetate, whose share in the analyzed samples was 4.48–21.36%. Other terpene compounds found in all samples are limonene, *β*-selinene, *α*-copaene, italicene, *α*-terpineol, *Ar*-curcumene, and *β*-selinene. Eight out of nine obtained EOs could be classified as high quality considering the proportions of neryl acetate and α-pinene.

Obtained EOs showed moderate or low cytotoxic potential in three cell lines: skin (fibroblast), lung, and breast cancer. The lung cell line was the least resistant to EOs, while the breast cancer cell line was the most resistant of the three cell lines used. To gain insight into which compounds or compound groups impact EO’s cytotoxic potential, we performed a statistical analysis, which led to the identification of several compounds or compound groups with significant activity for the individual cell line. As EOs are complex mixtures, we simulated *H. italicum* EO using five pure compounds: linalool, *α*-terpineol, terpinene-4-ol, nerol, and nerolidol. The results showed a conclusive antagonistic effect on their individual cytotoxic potential, more evident in the MDA-MB-231 cell line, in which individual compounds were more toxic than in the HDF cell line. These results explain why this cell line was the most resistant to *H. italicum* EO. Furthermore, this study supports the idea of using *H. italicum* in the human diet, indicating its safety for dietary consumption.

## Figures and Tables

**Table 1:** Chemical composition of *H. italicum* EOs (amount of compounds is expressed in %).

Compound	RI-E	RI-L	LA-P	LA-Z	VI	KS	PL	PT	MA	KO-1	KO-2	ID
***α*-Pinene**	940	943	12.64	11.28	8.45	4.25	9.99	12.49	7.41	7.30	4.01	RI, MS
*α*-Fenchene	953	954	0.00	0.00	0.00	0.00	0.00	0.33	0.00	0.00	0.00	RI, MS
**Limonene**	1033	1035	3.60	5.16	1.41	1.96	1.97	5.74	2.76	3.70	2.44	RI, MS
*1,8*-Cineole	1036	1036	0.67	0.00	0.00	0.00	0.00	0.78	0.00	0.00	0.00	RI, MS
Isobutyl angelate	1054	1055	0.69	0.00	0.57	0.00	0.00	0.73	0.00	0.00	0.00	RI, MS
Linalool	1100	1103	1.02	nd	1.47	1.05	0.87	2.17	1.03	1.49	1.39	RI, MS
***2*-Methylbutyl angelate**	1156	1158	1.42	1.11	3.27	0.83	0.98	2.89	2.23	3.41	2.17	RI, MS
*endo*-Borneol	1170	1171	0.00	1.81	0.00	0.00	0.00	0.00	0.00	0.00	0.00	RI, MS
Terpinen-*4*-ol	1180	1181	0.00	0.00	0.00	0.00	0.00	0.00	0.00	1.21	0.87	RI, MS
**4,6-Dimethyloctane-3,5-dione**	1188	-	1.75	3.44	2.43	1.95	2.64	3.61	2.26	1.97	2.35	RI
***α*-Terpineol**	1193	1195	1.79	2.60	1.01	1.55	1.82	1.65	1.35	1.73	1.73	RI, MS
Nerol	1231	1234	0.00	0.00	0.95	0.84	1.76	1.67	1.76	3.49	2.74	RI, MS
**Neryl acetate**	1367	1371	10.63	4.48	20.00	11.00	15.50	17.60	21.36	10.97	11.12	RI, MS
***α*-Copaene**	1376	1378	2.47	3.10	0.48	2.70	2.77	1.70	1.99	2.90	2.95	RI, MS
**Italicene**	1402	1405	2.30	3.60	1.80	3.09	3.77	3.51	2.63	3.73	3.86	RI, MS
*cis-α*-Bergamotene	1414	1417	0.00	0.00	0.00	0.00	0.97	0.00	0.00	0.00	0.78	RI, MS
*trans-β*-Caryophyllene	1419	1421	1.72	2.08	0.90	0.00	1.72	2.86	3.07	2.30	2.54	RI, MS
*trans-α*-Bergamotene	1436	1437	0.00	0.00	0.00	0.81	1.03	0.00	0.00	0.00	0.85	RI, MS
**Italidione I**	1443	1446	9.17	2.75	2.37	2.50	4.37	3.54	2.24	4.07	3.61	RI, MS
Neryl propanoate	1455	1458	0.00	0.00	3.02	2.10	3.03	1.76	1.56	3.25	3.03	RI, MS
*γ*-Selinene	1475	1477	0.00	1.48	1.15	1.32	1.60	0.00	0.00	0.00	1.25	RI, MS
*γ*-Curcumene	1480	1480	0.00	0.00	0.00	0.00	1.53	1.11	3.00	6.85	6.82	RI, MS
***Ar*-curcumene**	1484	1487	5.92	7.01	8.14	10.65	7.57	9.21	6.54	8.08	7.07	RI, MS
***β*-Selinene**	1486	1488	6.88	4.85	6.85	2.27	6.44	2.42	6.89	5.25	5.21	RI, MS
**Italidione II**	1490	1493	10.90	8.86	6.16	4.23	5.10	2.84	4.71	5.56	4.68	RI, MS
*α*-Selinene	1494	1496	3.95	4.77	6.06	2.96	4.70	0.00	4.07	3.64	3.38	RI, MS
*δ*-Cadinene	1523	1525	1.07	1.15	0.00	0.00	1.19	0.00	1.22	1.12	1.30	RI, MS
Phenylethyl tiglate	1540	1541	0.00	0.00	0.00	1.51	0.00	0.00	0.00	0.00	0.92	RI, MS
Nerolidol	1566	1567	0.97	6.46	0.00	0.00	0.00	0.00	0.00	0.00	0.00	RI, MS
**Italidione III**	1582	1583	9.54	10.78	13.79	7.36	8.35	10.14	5.45	6.73	5.20	RI, MS
Viridiflorol	1594	1591	0.00	0.00	0.00	1.53	0.00	0.00	0.00	0.00	0.00	RI
Guaiol	1597	1599	0.00	0.00	0.00	0.00	0.00	1.37	0.00	0.00	1.08	RI, MS
Humulene epoxide II	1605	1605	3.22	4.47	0.00	5.87	2.28	1.07	3.67	2.14	3.16	RI
Rosifoliol	1608	1611	1.14	0.00	0.77	1.01	1.71	4.13	6.24	4.67	3.54	RI, MS
*γ*-Eudesmol	1634	1634	1.15	0.00	0.00	0.00	0.65	1.19	0.00	0.00	0.74	RI
*τ*-Cadinol	1643	1644	1.26	1.44	0.00	1.48	0.00	0.00	0.00	0.00	0.00	RI, MS
*β*-Eudesmol	1651	1654	1.17	0.00	0.00	1.84	1.13	1.82	0.00	0.00	1.63	RI, MS
*α*-Muurolol	1656	1654	2.06	2.85	2.15	3.40	3.19	0.00	3.17	2.25	2.87	RI, MS
*β*-Bisabolol	1671	1672	0.00	0.00	0.00	0.22	0.00	0.00	0.00	0.00	1.28	RI, MS
Neryl hexanoate	1729	1731	0.00	0.00	0.60	0.00	1.36	0.00	0.00	0.00	0.00	RI
	**TOTAL**		99.10	95.53	93.80	80.28	99.99	98.33	96.61	97.81	96.57	

RI-E-retention index determined on a HP-5MS column using the homologous series of *n*-alkanes (C_9_–C_25_); RI-L-retention index according to literature; locations (LA-Lastovo, VI-Vitina; KS-KaštelStari, PL-Plano, PT-Prgomet, KO-Kornati) ID-manner of identification of compounds; MS mass spectra.

**Table 2:** Estimated IC_50_ (=mean ± standard deviation (mg/L)) values after treatment of cells with essential oils of *H. italicum*.

Sample	HDF	BEAS-2B	MDA-MB-231
LA-P	184.77 ± 4.63	52.90 ± 1.15	212.83 ± 20.43
LA-Z	177.40 ± 1.44	59.97 ± 4.32	169.00 ± 3.61
VI	189.63 ± 4.82	162.33 ± 4.21	207.33 ± 13.04
KS	167.67 ± 2.89	150.00 ± 10.54	165.20 ± 1.59
PL	189.23 ± 14.35	104.67 ± 17.47	192.17 ± 12.09
PT	186.33 ± 6.90	173.67 ± 1.53	193.67 ± 6.60
MA	175.67 ± 4.54	112.50 ± 12.76	227.50 ± 28.81
KO-1	224.37 ± 20.35	90.20 ± 11.70	228.50 ± 34.65
KO-2	190.27 ± 6.51	55.80 ± 0.72	199.07 ± 15.08

**Table 3:** The results of statistical correlation analysis for cytotoxic activity and *H. italicum* EO’s chemical composition.

**Terpene Compounds vs. IC_50_**	**HDF**	**BEAS-2B**	**MDA-MB-213**
Monoterpene hydrocarbons	ns	ns	ns
Derivatives of monoterpenes	ns	*r* = 0.5948 *p* = 0.0456	ns
Sesquiterpene hydrocarbons	ns	ns	ns
Derivatives of sesquiterpenes	ns	ns	ns
Other compounds	ns	ns	ns
**Functional Groups vs. IC_50_**	**HDF**	**BEAS-2B**	**MDA-MB-213**
Alkenes	ns	*r* = −0.7103 *p* = 0.0160	ns
Alcohols	ns	ns	ns
Ketones	ns	ns	ns
Aromatic	ns	*r* = 0.6173 *p* = 0.0383	ns
Esters	ns	*r* = 0.6547 *p* = 0.0278	ns
Other compounds	ns	ns	ns
**Size (Molecular Weight) vs. IC_50_**	**HDF**	**BEAS-2B**	**MDA-MB-213**
136.23	ns	ns	ns
150–197	ns	*r* = 0.6151 *p* = 0.0390	*r* = 0.5845 *p* = 0.0492
202–211	ns	*r* = −0.5953 *p* = 0.0454	ns
220–225	ns	*r* = −0.7598 *p* = 0.0088	ns
>225	ns	ns	ns
**Structure vs. IC_50_**	**HDF**	**BEAS-2B**	**MDA-MB-213**
Acylic (*n* = 0)	ns	ns	ns
Cyclic (*n* = 1)	ns	ns	ns
Cyclic (*n* = 2)	ns	*r* = −0.7733 *p* = 0.0073	ns
Cyclic (*n* = 3)	ns	ns	ns
**No. of Oxygen Atoms vs. IC_50_**	**HDF**	**BEAS-2B**	**MDA-MB-213**
*n* = 0	ns	*r* = −0.6407 *p* = 0.0315	ns
*n* = 1	ns	ns	ns
*n* = 2	ns	ns	ns
**No. of Unsaturated Bonds vs. IC_50_**	**HDF**	**BEAS-2B**	**MDA-MB-213**
*n* = 0	ns	ns	*r* = −0.7974 *p* = 0.0050
*n* = 1	ns	ns	ns
*n* = 2	ns	*r* = −06772 *p* = 0.0225	ns
*n* = 3	ns	ns	*r* = 0.6433 *p* = 0.0308
**Individual Compounds vs. IC_50_**	**HDF**	**BEAS-2B**	**MDA-MB-213**
2-Methylbutyl angelate	*r* = 0.6569 *p* = 0.0273	ns	*r* = 0.6606 *p* = 0.0264
Terpinen-4-ol	*r* = 0.8019 *p* = 0.0047	ns	ns
Nerol	*r* = 0.7088 *p* = 0.0163	ns	ns
*γ*-Curcumene	*r* = 0.6702 *p* = 0.0241	ns	ns
*α*-Terpineol	ns	*r* = −0.6237 *p* = 0.0363	ns
Neryl acetate	ns	*r* = 0.6496 *p* = 0.0292	ns
*α*-Copaene	ns	*r* = −0.06936 *p* = 0.0191	ns
Ar-curcumene	ns	*r* = 0.7437 *p* = 0.0108	ns
Italidione II	ns	*r* = −0.6532 *p* = 0.0282	ns
*δ*-Cadinene	ns	*r* = −0.8730 *p* = 0.0011	ns
*γ*-Selinene	ns	ns	*r* = −0.7128 *p* = 0.0156
*β*-Selinene	ns	ns	*r* = 0.6186 *p* = 0.0379
Rosifoliol	ns	ns	*r* = 0.6751 *p* = 0.0230

No—number; *r*—Pearson’s correlation coefficients; *p*—value of significance; ns—not statistically significant. Only statistically significant results (*p* < 0.05) were presented in the table.

**Table 4:** Locations of *H. italicum* plants sampling.

Name of Location	Latitude	Longitude	Approximate Elevation
Lastovo (LA-P)	42°45′23″ N	16°54′48″ E	31 m
Lastovo (LA-Z)	42°44′44″ N	16°52′21″ E	120 m
Vitina (VI)	43°14′39″ N	17°28′52″ E	174 m
Kaštel Stari (KS)	43°34′49″ N	16°19′40″ E	429 m
Plano (PL)	43°33′52″ N	16°16′58″ E	263 m
Prgomet (PT)	43°37′04″ N	16°14′43″ E	337 m
Marina (MA)	43°30′33″ N	16°07′49′′ E	18 m
Kornati (KO-1, KO-2)	43°49′30″ N	15°16′19″ E	38 m

## Data Availability

Not applicable.

## Supplementary Materials

The following supporting information can be downloaded at: https://www.mdpi.com/article/10.3390/molecules28145299/s1. Figure S1: Chemical composition of essential oil of *Helichrysum italicum* (Roth) G. Don; Table S1: Classification of identified compounds according to different groups; Table S2: The results of statistical correlation analysis for cytotoxic activity and different groups of compounds; Table S3: The results of statistical correlation analysis for cytotoxic activity and compounds identified in *H. italicum* EOs.

## Sample Availability

Samples of the compounds and plant material are available from the authors.
