# Supplementary material for: Chemical Profiling and Bioactivity Assessment of Helichrysum italicum (Roth) G. Don. Essential Oil: Exploring Pure Compounds and Synergistic Combinations
Source: Molecules. 2023 Jul 9;28(14):5299. doi: 10.3390/molecules28145299 (PMC10384098; doi:10.3390/molecules28145299)
Supplement: Supplementary file 1 [file molecules-28-05299-s001.zip › molecules-2493480-supplementary.pdf]

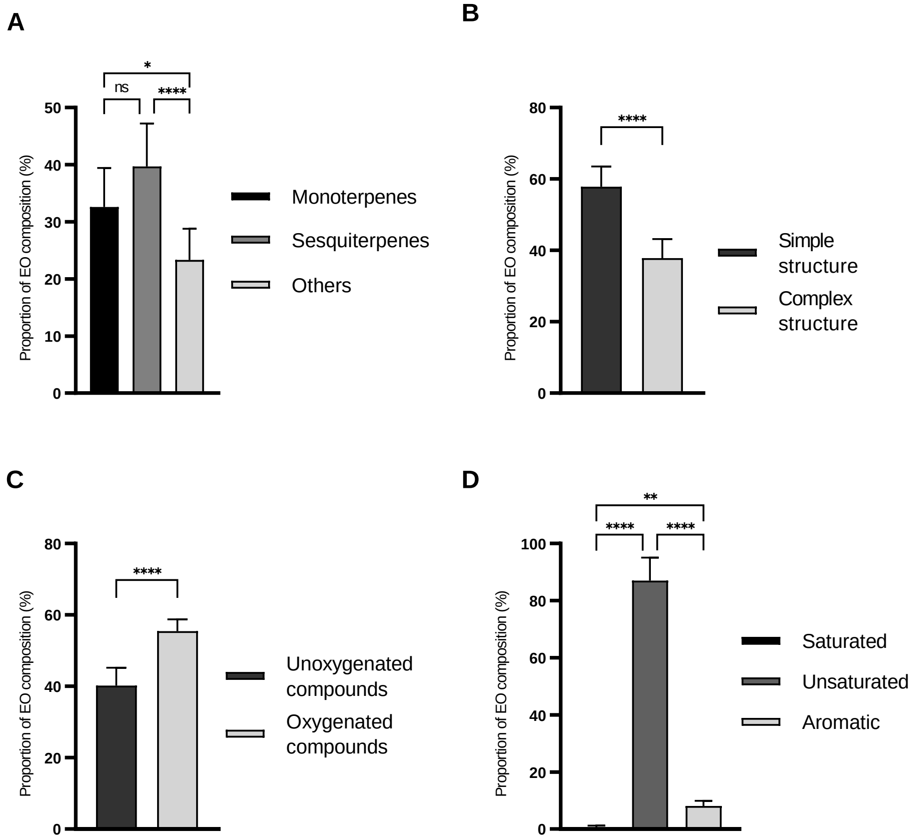

Figure S1. Chemical composition of essential oil of *Helichrysum italicum* (Roth) G. Don **(A)** Proportion of monoterpene, sesquiterpene, and other compounds in *H. italicum* EOs. **(B)** Proportion of compounds with simple (non or one carbon ring in the structure) and complex (two or more carbon rings) structure. **(C)** Proportion of unoxygenated and oxygenated compounds in the EOs composition. **(D)** Proportion of saturated, unsaturated, and aromatic compounds in the EOs composition. Values were compared by performing a two-way ANOVA test with post hoc Tukey's corrected multiple comparison tests for charts A and D, while a two-tailed unpaired *t*-test was used for charts B and C. Symbols for different test significance levels are assigned as follows: not significant (*ns*) for $p \geq 0.05$, * for $0.01 \leq p < 0.05$, ** for $0.001 \leq p < 0.01$, *** for $0.0001 \leq p < 0.001$, and **** for $p < 0.0001$. All data are presented as mean ± standard deviation (SD).

Table S1. Classification of identified compounds according to different groups. The amount of compounds for each group in EO's is expressed in %. *Ar*-aromatic compounds.

**Terpene Compounds**	**LA-P**	**LA-Z**	**VI**	**KS**	**PL**	**PT**	**MA**	**KO-1**	**KO-2**
Monoterpene hydrocarbons	16.24	16.44	9.86	6.21	11.96	18.56	10.17	11.00	6.45
Derivatives of monoterpenes	14.11	8.89	27.05	16.54	24.34	25.63	27.06	22.14	20.88
Sesquiterpene hydrocarbons	24.31	28.04	25.38	23.80	33.29	20.81	29.41	33.87	36.01
Derivatives of sesquiterpenes	10.97	15.22	2.92	15.35	8.96	9.58	13.08	9.06	14.30
Others	33.47	26.94	28.59	18.38	21.44	23.75	16.89	21.74	18.93
**Functional Groups**	**LA-P**	**LA-Z**	**VI**	**KS**	**PL**	**PT**	**MA**	**KO-1**	**KO-2**
Alkenes	34.63	37.47	27.10	19.36		30.16	33.04	36.79	35.39
Alcohols	10.56	15.16	6.35	12.92	11.13	14.00	13.55	14.84	17.87
Ketone	31.36	25.83	24.75	16.04	20.46	20.13	14.66	18.33	15.84
Aromatics	5.92	7.01	8.14	12.16	7.57	9.21	6.54	8.08	7.99
Esters	12.74	5.59	27.46	13.93	20.87	22.98	25.15	17.63	16.32
Others	3.89	4.47	0.00	5.87	2.28	1.85	3.67	2.14	3.16
**Size (Molecular weight)**	**LA-P**	**LA-Z**	**VI**	**KS**	**PL**	**PT**	**MA**	**KO-1**	**KO-2**
136.23	16.24	16.44	9.86	6.21	11.96	18.56	10.17	11.00	6.45
150-197	17.97	13.44	29.70	17.22	23.57	31.10	29.99	24.27	22.37
202-211	33.48	30.79	30.77	29.91	40.69	26.11	33.21	41.19	43.57
220-225	21.87	24.08	9.08	19.58	14.06	12.42	17.79	14.62	18.98
>225	9.54	10.78	14.39	7.36	9.71	10.14	5.45	6.73	5.20
**Structure**	**LA-P**	**LA-Z**	**VI**	**KS**	**PL**	**PT**	**MA**	**KO-1**	**KO-2**
Acyclic	46.09	37.88	54.63	31.86	43.96	46.95	42.60	40.94	36.29
Cyclic (1)	11.31	14.77	10.56	15.89	12.89	17.71	13.65	21.57	21.13
Cyclic (2)	36.93	36.18	26.33	25.21	36.60	28.46	35.74	28.67	32.34
Cyclic (3)	4.77	6.70	2.28	7.32	6.54	5.21	4.62	6.63	6.81
**Number of Oxygen Atoms**	**LA-P**	**LA-Z**	**VI**	**KS**	**PL**	**PT**	**MA**	**KO-1**	**KO-2**
n=0	40.55	44.48	35.24	30.01	45.25	39.37	39.58	44.87	42.46

n=1	14.45	19.63	6.35	18.79	13.41	15.85	17.22	16.98	21.03
n=2	44.10	31.42	52.21	31.48	41.33	43.11	39.81	35.96	33.08

**Unsaturated Bonds in Molecules**	**LA-P**	**LA-Z**	**VI**	**KS**	**PL**	**PT**	**MA**	**KO-1**	**KO-2**
n=0	0.67	1.81	0.00	1.53	0.00	0.78	0.00	0.00	0.00
n=1	25.98	24.87	14.66	19.32	25.03	28.19	22.79	23.79	23.28
n=2	25.32	28.51	25.06	20.08	28.15	23.16	28.96	28.51	30.84
n=3	41.21	33.33	45.94	27.19	39.24	36.99	38.32	37.43	34.46
Ar	5.92	7.01	8.14	12.16	7.57	9.21	6.54	8.08	7.99

Table S2. The results of statistical correlation analysis for cytotoxic activity and different groups of compounds. *No*-number, *r*-Pearson's correlation coefficients; *p*-value of significance; *ns*-not statistically significant. Only statistically significant results ($p < 0.05$) were presented in the table.

**Terpene compounds vs. IC$_{50}$**	**HDF**	**BEAS-2B**	**MDA-MB-213**
Monoterpene hydrocarbons	r = 0.02734<br>p = 0.4722	r = -0.0472<br>p = 0.4520	r = -0.0163<br>p = 0.4834
Derivatives of monoterpenes	r = 0.2639<br>p = 0.2463	***r* = 0.5948**<br>***p* = 0.0456**	r = 0.5515<br>p = 0.0619
Sesquiterpenes	r = 0.4945<br>p = 0.0880	r = -0.5753<br>p = 0.0525	r = 0.3008<br>p = 0.2158
Derivatives of sesquiterpenes	r = -0.4068<br>p = 0.1386	r = -0.5008<br>p = 0.0848	r = -0.4046<br>p = 0.1401
Other compounds	r = 0.06804<br>p = 0.4310	r = -0.1932<br>p = 0.3092	r = 0.0235<br>p = 0.4761
**Functional groups vs. IC$_{50}$**	**HDF**	**BEAS-2B**	**MDA-MB-213**
Alkenes	r = 0.4760<br>p = 0.0976	***r* = -0.7103**<br>***p* = 0.0160**	r = 0.3677<br>p = 0.1651
Alcohols	r = 0.1030<br>p = 0.3960	r = -0.4751<br>p = 0.0981	r = -0.1055<br>p = 0.3935
Ketones	r = -0.0125<br>p = 0.4873	r = -0.2793<br>p = 0.2333	r = -0.0537<br>p = 0.4454
Aromatics	r = -0.2095<br>p = 0.2943	***r* = 0.6173**<br>***p* = 0.0383**	r = -0.5549<br>p = 0.0605
Esters	r = 0.1575<br>p = 0.3429	***r* = 0.6547**<br>***p* = 0.0278**	r = 0.5369<br>p = 0.0680
Other compounds	r = -0.5523<br>p = 0.0615	r = -0.3534<br>p = 0.1755	r = -0.4858<br>p = 0.0925

**Structure vs. IC_{50}**	**HDF**	**BEAS-2B**	**MDA-MB-213**
Acylic (n = 0)	*r* = 0.2294	*r* = 0.3504	*r* = 0.4752
	p = 0.2764	*p* = 0.1776	*p* = 0.0981
Cyclic (n = 1)	*r* = 0.5045	*r* = -0.1634	*r* = 0.0592
	p = 0.0830	*p* = 0.3372	*p* = 0.4398
Cyclic (n = 2)	*r* = -0.1277	***r* = -0.7733**	*r* = 0.1376
	p = 0.3717	***p* = 0.0073**	*p* = 0.3621
Cyclic (n = 3)	*r* = 0.0251	r = -0.3848	*r* = -0.4601
	p = 0.4744	p = 0.1532	*p* = 0.1064

**Size (molecular weight) vs. IC_{50}**	**HDF**	**BEAS-2B**	**MDA-MB-213**
136.23	*r* = 0.0273	r = -0.0472	*r* = -0.0163
	p = 0.4722	p = 0.4520	*p* = 0.4834
150-197	*r* = 0.2445	***r* = 0.6151**	***r* = 0.5845**
	p = 0.2630	***p* = 0.0390**	***p* = 0.0492**
202-211	*r* = 0.5658	***r* = -0.5953**	*r* = 0.3569
	p = 0.0561	***p* = 0.0454**	*p* = 0.1729
220-225	*r* = -0.3691	***r* = -0.7598**	*r* = -0.3337
	p = 0.1642	***p* = 0.0088**	*p* = 0.1901
>225	*r* = -0.0756	r = 0.3670	*r* = -0.2348
	p = 0.4234	p = 0.1657	*p* = 0.2716

**No. of oxygen atoms vs. IC_{50}**	**HDF**	**BEAS-2B**	**MDA-MB-213**
n = 0	*r* = 0.5391	***r* = -0.6407**	*r* = 0.2991
	p = 0.0671	***p* = 0.0315**	*p* = 0.2171
n = 1	*r* = -0.1113	*r* = -0.5084	*r* = -0.2563
	p = 0.3878	*p* = 0.0811	*p* = 0.2528
n = 2	*r* = 0.1156	*r* = 0.4325	*r* = 0.4473
	p = 0.3836	*p* = 0.1225	*p* = 0.1137

**No. of unsaturated bonds vs. IC_{50}**	**HDF**	**BEAS-2B**	**MDA-MB-213**
n = 0	*r* = -0.5503	*r* = -0.0114	***r* = -0.7974**
	p = 0.0623	*p* = 0.4883	***p* = 0.0050**
n = 1	*r* = 0.1279	*r* = -0.4573	*r* = 0.0192
	p = 0.3715	*p* = 0.1080	*p* = 0.4804
n = 2	*r* = 0.3978	***r* = -06772**	*r* = 0.4341
	p = 0.1445	***p* = 0.0225**	*p* = 0.1215
n = 3	*r* = 0.3544	*r* = 0.0724	***r* = 0.6433**
	p = 0.1747	*p* = 0.4266	***p* = 0.0308**

Table S3. The results of statistical correlation analysis for cytotoxic activity and compounds identified in *H. italicum* EOs. *No*-number, *r*-Pearson's correlation coefficients; *p*-value of significance; *ns*-not statistically significant. Only statistically significant results ($p < 0.05$) were presented in the table.

Compound	HF77FA		BEAS-2B		MDA-MB-231	
	r	*p*	*r*	*p*	*r*	*p*
α-Pinene	-0.0019	0.4981	-0.0277	0.4718	0.0313	0.4681
α-Fenchene	-0.0220	0.4776	0.5364	0.0683	-0.0966	0.4023
Limonene	0.0862	0.4127	-0.1185	0.3807	-0.1089	0.3901
1,8-Cineole	-0.0587	0.4404	0.1328	0.3667	0.0765	0.4225
Isobutyl angelate	-0.0246	0.4749	0.3452	0.1815	0.1570	0.3433
Linalool	0.3033	0.2326	0.4650	0.1228	0.0300	0.4719
2-Methylbutyl angelate	**0.6569**	**0.0273**	0.3315	0.1918	**0.6606**	**0.0264**
endo-Borneol	-0.2328	0.2733	-0.3769	0.1587	-0.5074	0.0816
Terpinen-4-ol	**0.8019**	**0.0047**	-0.3712	0.1627	0.4137	0.1342
4,6-Dimethyloctane-3,5-dione	-0.1807	0.3209	0.2385	0.2683	-0.4268	0.1260
α-Terpineol	-0.0415	0.4578	**-0.6237**	**0.0363**	-0.4686	0.1016
Nerol	**0.7088**	**0.0163**	0.0186	0.4811	0.5025	0.0840
Neryl acetate	-0.0461	0.4532	**0.6496**	**0.0292**	0.4968	0.0868
α-Copaene	0.0832	0.4157	**-0.6936**	**0.0191**	-0.2763	0.2359
Italicene	0.2791	0.2335	-0.2855	0.2282	-0.2871	0.2269
cis-α-Bergamotene	0.0862	0.4127	-0.2894	0.2251	-0.1057	0.3934
trans-β-Caryophyllene	0.2872	0.2268	-0.2874	0.2267	0.5094	0.0807
trans-α-Bergamotene	-0.1963	0.3063	-0.0640	0.4351	-0.4373	0.1196
Italidione I	0.1765	0.3249	-0.4985	0.0860	0.2558	0.2532
Neryl propanoate	0.5067	0.0820	0.3541	0.1749	0.2450	0.2626
γ-Selinene	-0.3331	0.1905	-0.0597	0.4394	**-0.7128**	**0.0156**
γ-Curcumene	**0.6702**	**0.0241**	-0.3537	0.1752	0.5128	0.0790
Ar-curcumene	-0.1175	0.3817	**0.7437**	**0.0108**	-0.4981	0.2481
β-Selinene	0.2077	0.2959	-0.4268	0.1260	**0.6186**	**0.0379**
Italidione II	-0.0301	0.4693	**-0.6532**	**0.0282**	0.0373	0.4621
α-Selinene	0.0323	0.4671	-0.3125	0.2065	0.1250	0.3743
δ-Cadinene	0.2621	0.2479	**-0.8730**	**0.0011**	0.3449	0.1817
2-Phenylethyl tiglate	-0.3797	0.1567	0.0872	0.4118	-0.5206	0.0754
Nerolidol	-0.2435	0.2639	-0.4454	0.1148	-0.4776	0.0968
Italidione III	-0.0916	0.4073	0.3591	0.1713	-0.2380	0.2688
Viridiflorol	-0.4625	0.1050	0.3463	0.1807	-0.5706	0.0543
Guaiol	0.0285	0.4709	0.1784	0.3231	-0.0854	0.4135
Humulene epoxide II	-0.5242	0.0737	-0.3665	0.1660	-0.4844	0.0932
Rosifoliol	0.3338	0.1900	0.0915	0.4074	**0.6751**	**0.0230**

γ-Eudesmol	-0.0070	0.4929	-0.1127	0.3864	0.0363	0.4630
τ-Cadinol	-0.2867	0.2273	-0.5653	0.0564	-0.4234	0.1281
β-Eudesmol	-0.3153	0.2043	0.1661	0.3347	-0.4439	0.1157
α-Muurolol	-0.2511	0.2573	-0.3947	0.1466	-0.1569	0.3434
β-Bisabolol	-0.0085	0.4914	-0.3533	0.1755	-0.1055	0.3935
Neryl hexanoate	0.0684	0.4306	0.1715	0.3295	-0.0614	0.4377
